# Field Efficacy of Vectobac GR as a Mosquito Larvicide for the Control of Anopheline and Culicine Mosquitoes in Natural Habitats in Benin, West Africa

**DOI:** 10.1371/journal.pone.0087934

**Published:** 2014-02-05

**Authors:** Armel Djènontin, Cédric Pennetier, Barnabas Zogo, Koffi Bhonna Soukou, Marina Ole-Sangba, Martin Akogbéto, Fabrice Chandre, Rajpal Yadav, Vincent Corbel

**Affiliations:** 1 Faculté des Sciences et Techniques/MIVEGEC (IRD 224-CNRS 5290-UM1-UM2), Université d’Abomey Calavi/Centre de Recherche Entomologique de Cotonou (CREC), Cotonou, Bénin; 2 MIVEGEC (IRD 224-CNRS 5290-UM1-UM2), Centre de Recherche Entomologique de Cotonou (CREC), Cotonou, Bénin; 3 Faculté des Sciences et Techniques/Centre de Recherche Entomologique de Cotonou (CREC), Université d’Abomey Calavi/Centre de Recherche Entomologique de Cotonou (CREC), Cotonou, Bénin; 4 MIVEGEC (IRD 224-CNRS 5290-UM1-UM2), Laboratoire de lutte contre les Insectes Nuisibles (LIN), Montpellier, France; 5 Department of Control of Neglected Tropical Diseases, World Health Organization, Geneva, Switzerland; 6 MIVEGEC (IRD 224-CNRS 5290-UM1-UM2)/Department of Entomology, Kasetsart University, Ladyaow Chatuchak Bangkok, Thailand; Université Pierre et Marie Curie, France

## Abstract

**Introduction:**

The efficacy of Vectobac GR (potency 200 ITU/mg), a new formulation of bacterial larvicide *Bacillus thuringiensis var. israelensis* Strain AM65-52, was evaluated against *Anopheles gambiae* and *Culex quinquefasciatus* in simulated field and natural habitats in Benin.

**Methods:**

In simulated field conditions, Vectobac GR formulation was tested at 3 dosages (0.6, 0.9, 1.2 g granules/m^2^ against *An. gambiae* and 1, 1.5, 2 g granules/m^2^ against *Cx. quinquefasciatus*) according to manufacturer’s product label recommendations. The dosage giving optimum efficacy under simulated field conditions were evaluated in the field. The efficacy of Vectobac GR in terms of emergence inhibition in simulated field conditions and of reduction of larval and pupal densities in rice fields and urban cesspits was measured following WHO guidelines for testing and evaluation of mosquito larvicides.

**Results:**

Vectobac GR caused emergence inhibition of ≥80% until 21 [20]–[22] days for *An. gambiae* at 1.2 g/m^2^ dose and 28 [27–29] days for *Cx. quinquefasciatus* at 2 g/m^2^ in simulated field habitats. The efficacy of Vectobac GR in natural habitats was for 2 to 3 days against larvae and up to 10 days against pupae.

**Conclusions:**

Treatment with Vectobac GR caused complete control of immature mosquito within 2–3 days but did not show prolonged residual action. Larviciding can be an option for malaria and filariasis vector control particularly in managing pyrethroid-resistance in African malaria vectors. Since use of larvicides among several African countries is being emphasized through Economic Community of West Africa States, their epidemiological impact should be carefully investigated.

## Introduction

Malaria in Sub-Saharan Africa is a major public health problem accounting for 79% of global incidence of cases and 90% of deaths [1]. Lymphatic filariasis is a widely prevalent neglected vector-borne disease in Africa [2]. While chemotherapy for malaria control and mass drug administration against filariasis have been extensively used in disease endemic countries, vector control can complement strategies for prevention and control of these diseases [3]. Complementary vector control tools targeting exophagic and exophilic vectors or targeting another stage in the mosquito’s lifecycle (e.g. the aquatic stage) are then needed to achieving the Millennium Development Goals for malaria control by 2015 [4].

Larval source management is an important component of an integrated vector management approach [5] and has extensively been used for the control of anophelines since the 1950s [6]. Recent studies in rural areas of Eastern Africa demonstrated that larval control by hand application of larvicides can reduce the abundance of malaria mosquito larvae and adults and transmission by 70–90% where the majority of aquatic mosquito larval habitats are accessible and relatively limited in number and size [7]. Larval source management offers the dual benefits of reducing numbers of house-frequenting mosquitoes and those that bite outdoors.

Larviciding is a commonly used method of mosquito control in different ecological patterns mostly in urban areas or some coastal areas where breeding sites are well identified. Currently 10 formulations are recommended by WHOPES for mosquito larval control, including microbial agents [8]. These bio-pesticides offer interesting prospects for the control of malaria vectors through varied and diverse groups of micro-organisms including viruses, bacteria and fungi which constitute an important part of the active ingredient arsenal for Integrated Vector Control [5].

*Bacillus thuringiensis israelensis (Bti)* and *Bacillus sphaericus (Bs)* have been extensively evaluated in the laboratory against anophelines and culicines larvae and also tested in a variety of environmental settings [9]. *Bti* is unlikely to pose any hazard to humans, other vertebrates and non-target invertebrates, provided that it is free from non-*Bt* microorganisms and biologically active products other than the insecticidal crystal proteins [10]. It was recently demonstrated that long-term use of *Bacillus thuringiensis israelensis* in coastal wetlands had no influence on the temporal evolution of the taxonomic structure and taxa abundance of non-target aquatic invertebrate communities [11]. In Benin, larviciding by the use of *Bti* was recently integrated as a part of vector management for malaria prevention [12].

While various *Bti* formulations are available as mosquito larvicides today, there has always been a need to improve them for better efficacy, ease of application and acceptability. In the present study in southern Benin, a new granular formulation of *Bti,* Vectobac GR of Valent BioSciences Corp, USA, was evaluated against *Anopheles gambiae* and *Culex quinquefasciatus* in simulated field experiments and in natural breeding habitats. The experimental procedures followed the WHO guidelines for testing and evaluation of mosquito larvicides [13]. The National Ethical Committee for Medical Research of Benin (N°006) cleared the study and the work was supervised by the WHO Pesticide Evaluation Scheme.

## Methods

### 1. Ethics Statement

Ethics clearance for the study was obtained from the National Ethical Committee for Medical Research in Benin (ethics clearance N°006 of 28^th^ April, 2011). The trial on *An. gambiae* was conducted after having received formal agreement from the president of local farmers named Lokossou Nestor. Concerning the trial on *Cx. quinquefasciatus*, permission from each owner of houses where cesspits were located was obtained before the trial was conducted.

### 2. Test Material

Vectobac GR is a new granular formulation of *Bacillus thuringiensis, subsp. israelensis*, strain AM65-52 developed by Valent BioSciences Corp., USA. The bio potency of this larvicide is 200 International Toxic Units (ITU)/mg product. Bio potency of products based on *Bti* is compared with a lyophilized reference powder (IPS82, strain1884) of this bacterial species using early fourth instar larvae of *A. aegypti* (strain Bora Bora). The potency of IPS82 has been arbitrarily designated as 15 000 ITU/mg powder against this strain of mosquito larva.

According to the manufacturer’s Material Safety Data Sheet, Vectobac GR is non-toxic by ingestion, skin contact or inhalation. It has no adverse effect on birds, earthworms, fish, or numerous other non-target aquatic invertebrates.

### 3. Mosquito Species

The Kisumu strain of *An. gambiae* and the F1 progeny *of* local population of *Culex quinquefasciatus* were used for the simulated field trial. Kisumu strain of *An. gambiae* is a reference strain maintained at the insectary of the Centre de Recherche Entomologique de Cotonou (CREC) and is free of any resistance mechanism.

### 4. Study Area

The simulated field trial was carried out in the Centre de Recherche Entomologique de Cotonou (CREC). The field trial with *An. gambiae* was conducted in a rice field located in Lélé, Covè district located in Department of Zou (7°13′ 8′′ N, 2°20′ 22′′ E). Concerning *Cx. quinquefasciatus*, the field trial was conducted in Cotonou, Department of Littoral (6°23N–2°25E).

### 5. Study Design

#### 5.1. Simulated field studies

The main objective of simulated field studies were to test and determine the optimum field application dosage of Vectobac GR. Vectobac GR was tested at 3 dosages against *An. gambiae* (0.6, 0.9, 1.2 g granules/m^2^) and *Cx. quinquefasciatus* (1, 1.5, 2 g granules/m^2^) according to manufacturer’s product label recommendations. Four replicates of the experiments were run for both treatments and control.

*Experimental set up:* Artificial cement containers (i.e. rectangular pits of 60 cm long × 30 cm width × 30 cm depth) were used to study the Vectobac GR dose-efficacy relation. Containers were half-filled with water and covered with a mosquito netting piece to prevent oviposition by wild female mosquitoes and the deposit of debris, and were placed under a shelter to prevent direct exposure of rain and sunlight.

*Bti application*: At t0, measured quantity of Vectobac GR was dispensed manually taking necessary safety precautions using gloves and facial masks.

*Cohort monitoring:* Larvicidal activity might last longer than the developmental period. In this context, cohort of 30 to 50 second instars larvae of *An. gambiae* or *Cx. qinquefasciatus* were released in each container every 7 to 10 days, depending on the larval development time frame. Each *An. gambiae* and *Cx. quinquefasciatus* larvae cohort was fed with 0.5 g and 1 g of cat food respectively when released. Each day after treatment, pupae were counted and removed from the containers and placed in plastic cups with water and covered with a netting piece. Temperature and pH of water in the containers were recorded daily and meteorological data were obtained from the National Meteorology Department. The studies were conducted between 8 June and 21 July 2011 with *An. gambiae* and between 10 August and 21 September 2011 with *Cx. quinquefasciatus*.

#### 5.2. Field trials

The field trial was launched with a formal agreement with the president of local rice field farmers. For the study, thirty ponds of 8 m^2^ (2 m×4 m) each were delimited with natural barrier made of local mud. Thirty cesspits with a surface ranging from 0.14 to 3.46 m^2^ housing *Cx. quinquefasciatus* larvae were selected in Cotonou and geo-referenced. All breeding sites were checked to confirm the presence of larvae before applying Vectobac GR.

One dose that provided the optimum efficacy in the simulated field studies was tested in natural habitats. Fifteen breeding sites of each type were treated with Vectobac while the remaining ones were left untreated and served as controls. Vectobac GR was uniformly applied manually on the water surface. Three replicates were run for each treatment or control corresponding to 45 treated habitats and 45 of untreated habitats.

Before treatment, each breeding site was sampled twice to determine the density of mosquito larvae and pupae. After treatment, sampling was done on days 1, 2, 3, and 7, and thereafter every third day until the density of larvae in the treated habitats reached to that of the control. The larval sampling method consisted of 3 dips using a ladle (350 ml). Sampling was done by the same operator. The larval instars as well as pupae were counted separately. Temperature and pH were monitored at each sampling day in each mosquito breeding sites. The field trials were conducted between 10 November and 23 December 2011 with *An. gambiae* and between 14 September and 5 November 2011 with *Cx. quinquefasciatus*.

### 6. Data Analysis

The data analyses were performed using R software (version 2.11.1). Data from the simulated field trial were used to estimate the Emergence Inhibition Rates (% EIR) for each treatment according to the following formula:
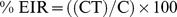
where C is the emergence rate in the control and T is the emergence rate in the treated containers at the same period of time [13].

A logistic regression model with a logit link was fitted to the data to investigate the effect of the treatments on the emergence rate. The influence explanatory covariables on the emergence rate was investigated by including in the models the dose, the number of day post-treatment and the replicates. The number of day after which the emergence rates significantly increased to more than 20% with 95% Confidence Intervals was estimated for each treatment based on the logistic regression model.

Concerning data from the field trials, the mean number of larvae and pupae collected (i.e. density) per sampling day was calculated for both treated and control groups. The first and second instars larvae (L1+L2) were pooled as early instars and the third and fourth instars (L3+L4) as late instars. Density Reduction (DR) of early and late instars larvae as well as pupae was estimated post-treatment using Mulla’s formula as follows:
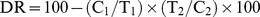
where C_1_ is the average number of larvae or pupae in control breeding sites prior to treatment and C_2_ is the average number of larvae in control breeding sites at each day of sampling. T_1_ is the average number of larvae or pupae in breeding sites to be treated with Vectobac GR and T_2_ is the average number of larvae or pupae in treated breeding sites for each sampling day [13]. When DR was negative (i.e. densities were higher in the treated group than the control group), the value was taken as zero.

Then, a linear regression model was fitted to the data to investigate the effect of the treatment on the density reduction. The influence of the time as explanatory covariable on the density reduction was investigated by including the time in the models. The number of day after which the density reduction reached 80% and 50% was then estimated.

## Results

### 1. Simulated Field Studies

The average temperature recorded in containers during trials with *An. gambiae* was 26.5°C (ranging from 24.0°C to 27.8°C) and 26.5°C (25.0°C to 27.7°C) with *Cx. quinquefasciatus*. The water pH was 7.5 (6.8 to 8.7) and 6.9 (6.6 to 8.0) for *An. gambiae* and *Cx. quinquefasciatus* containers, respectively.

Three thousands three hundred and sixty (3,360) larvae of *An. gambiae* were released in the containers for the trial. Emergence rates (ER) and Emergence Inhibition Rates (EIR) for each treatment are shown in Table 1. Emergence rates in the control ranged from 90% [86–94] to 96% [93–99]. The EIR were >80% for all dosages up to day 19 post-treatment. After day 26, the EIR was 44%, 55% and 63% at 0.6 g/m^2^, 0.9 g/m^2^ and 1.2 g/m^2^ doses of Vectobac GR, respectively. According to the logistic regression model, the estimated period of effectiveness (i.e. emergence rates <20%) was 15 days [14]–[17], 17 days [16]–[18] and 21 days [20]–[22] at the doses of 0.6, 0.9 and 1.2 g granule/m^2^, respectively.

**Table 1:** Emergence and Emergence Inhibition Rates (EIR) of *An. gambiae* larvae according to treatments.

N day post treatment		Control	0.6 g/m^2^	0.9 g/m^2^	1.2 g/m^2^
11	N	200	200	200	200
	NE	179	2	1	1
	ER (%) [95%CI]	90 [86–94]	1 [0–2]	1 [0–2]	1 [0–1]
	**EIR (%) [95%CI]**	**–**	**99**	**99**	**99**
19	N	200	200	200	200
	NE	180	31	27	17
	ER (%) [95%CI]	90 [86–94]	16 [11]–[21]	14 [9]–[19]	9 [5]–[13]
	**EIR (%) [95%CI]**	–	**83**	**85**	**91**
26	N	200	200	200	200
	NE	191	107	86	71
	ER (%) [95%CI]	96 [93–99]	54 [47–61]	43 [36–50]	36 [29–43]
	**EIR (%) [95%CI]**	**–**	**44**	**55**	**63**
35	N	120	120	120	120
	NE	114	105	104	72
	ER (%) [95%CI]	95 [91–99]	88 [82–94]	87 [81–94]	60 [51–69]
	**EIR (%) [95%CI]**	**–**	**8**	**9**	**37**
43	N	120	120	120	120
	NE	112	107	105	87
	ER (%) [95%CI]	93 [89–97]	89 [83–95]	88 [82–94]	73 [65–81]
	**EIR (%) [95%CI]**	**–**	**5**	**6**	**22**

*N = Number of larvae; NE = Number of larvae emerged; ER = Emergence Rate; EIR = Emergence Inhibition Rate.*

Three thousands seven hundred and sixty (3,760) larvae of *Cx. quinquefasciatus* were released in the containers during the simulated studies. Emergence and EIR for each treatment are shown in Table 2. Emergence rates in the control ranged from 90% [86–94] to 99% [98–100]. The EIR were 100% at day 11 regardless of the doses, and then decreased to 80% after 19 days of treatment with 1 g/m^2^ dose, 26 days at 1.5 g/m^2^ and 34 days at 2 g/m^2^. According to the logistic regression model, the estimated period of effectiveness (i.e. emergence rates <20%) was 19 days [18]–[20], 22 days [20]–[23] and 28 days [27–29] days at 1, 1.5 and 2 g granules/m^2^, respectively.

**Table 2:** Emergence and Emergence Inhibition Rates (EIR) of *Cx. quinquefasciatus* larvae according to the treatments.

N days post treatment		Control	1 g/m^2^	1.5 g/m^2^	2 g/m^2^
11	N	200	200	200	200
	NE	191	0	0	0
	ER [95%CI]%	96 [93–99]	0	0	0
	**EIR (%) [95%CI]%**	**–**	**100**	**100**	**100**
19	N	200	200	200	200
	NE	198	51	30	3
	ER [95%CI]%	99 [98–100]	26 [20–32]	15 [10]–[20]	02 [0–4]
	**EIR (%) [95%CI]%**	**–**	**74**	**85**	**99**
26	N	200	200	200	200
	NE	180	96	80	28
	ER [95%CI]%	90 [86–94]	48 [41–55]	40 [33–47]	14 [9]–[19]
	**EIR (%) [95%CI]%**	**–**	**47**	**56**	**84**
34	N	160	160	160	160
	NE	152	93	80	65
	ER [95%CI]%	95[92–98]	58[50–66]	50[42–58]	41[33–49]
	**EIR (%) [95%CI]%**		**38**	**47**	**57**
42	N	180	180	180	180
	NE	171	157	154	134
	ER [95%CI]%	95[92–98]	87[82–92]	86[81–91]	74[68–80]
	**EIR (%) [95%CI]%**		**8**	**10**	**22**

*N = Number of larvae; NE = Number of larvae emerged; ER = Emergence Rate; EIR = Emergence Inhibition Rate.*

### 2. Field Trials

Based on the results of simulated studies, doses that showed highest efficacies and residual activities against *An. gambiae* (1.2 g granules/m^2^) and *Cx. quinquefasciatus* (2 g granules/m^2^) were selected for the field trials.

The average temperature recorded in the breeding sites through the trial was 35.1°C (ranging from 28°C to 41.7°C) and 27.1°C (ranging from 25.1°C to 32.2°C) for *An. gambiae* and *Cx. quinquefasciatus,* respectively. The water pH was 6.6 (ranging from 5.1 to 8.8) and 6.8 (ranging from 5.7 to 8.1) in habitats with *An. gambiae* and *Cx. quinquefasciatus,* respectively. No rain was recorded during the trial on *An. gambiae* as the study was conducted during the dry season. The aerial average temperature recorded in the rice fields was 27.8°C, (23.2°C to 35.0°C). During the trial on *Cx. quinquefasciatus*, there was 345.90 mm rainfall while the average temperature was 27.3°C (24.6°C to 30.0°C).

**Figure 1:**
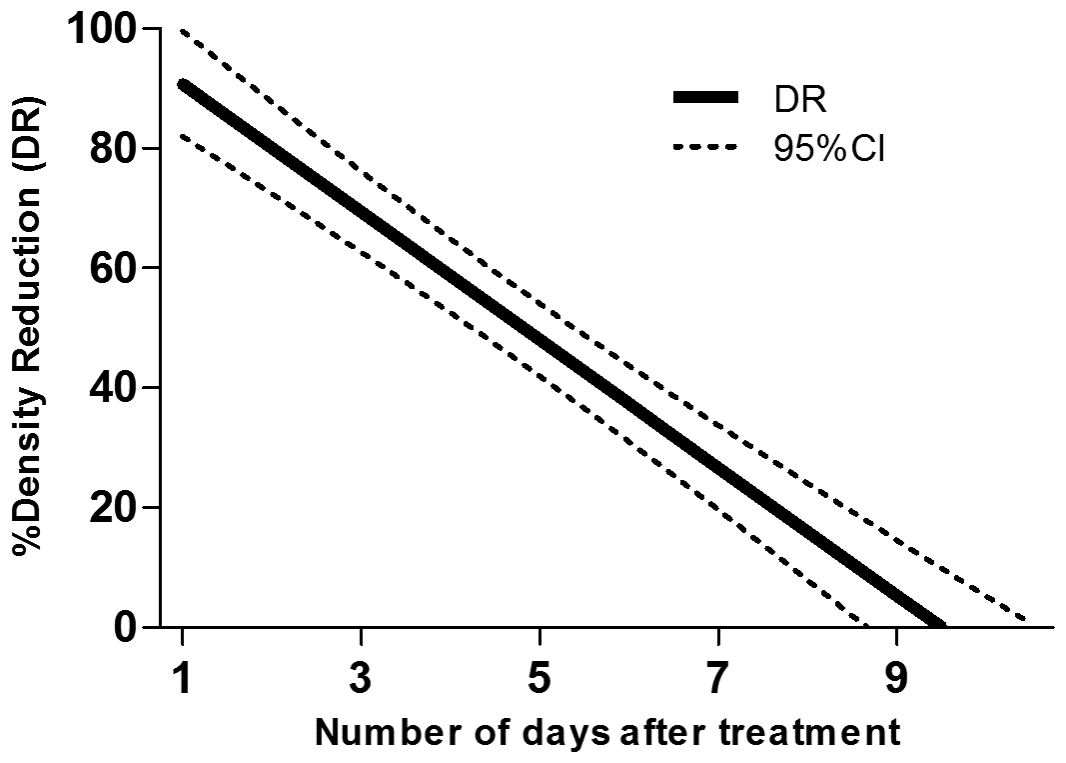
Density reduction (DR) of *An. gambiae* old instars larvae estimated by the regression model according to the number of days after treatment.

The mean number of *An. gambiae* larvae sampled per dip and the density reduction (DR) at each sampling day are shown in Table 3. Before treatment, mosquito larvae densities in the control ponds were 2.93 per dip, 0.56 per dip and 0.07 per dip for early instars larvae, late instars larvae and pupae respectively. In the ponds to be treated these densities were 3.02 per dip, 0.73 per dip and 0.04 per dip for early instars larvae, late instars larvae and pupae respectively. The highest efficacy of Vectobac GR in terms of reduction of early instars larvae of *An. gambiae* was observed three days post-treatment but was below 60% reduction. Vectobac GR reduced late instars larvae density by >80% up to 2 days post-treatment. The DR decreased to 73% after 3 days and to nil after day 7. The number of pupae was too low to make any comparisons between control and treated ponds. According to the logistic regression model, the estimated period for which the density of late instars larvae would be reduced by 80% (DR_80_) and 50% (DR_50_) was 2 days (1–3) and 5 days (4–6), respectively (Figure 1). The highest DR induced by Vectobac GR against early instars larvae was about 50%. Consequently The DR_80_ and DR_50_ values could not be estimated.

**Table 3:** Mean number of larvae and pupae per dip and density reduction (DR) after treatment Vectobac in natural habitats.

		*An. gambiae*								*Cx. quinquefasciatus*							
		Control				Treatment (1.2 g/m^2^)				Control				Treatment (2 g/m^2^)			
N day post treatment	Parameters	L1+L2	L3+L4	Pupae	Total	L1+L2	L3+L4	Pupae	Total	L1+L2	L3+L4	Pupae	Total	L1+L2	L3+L4	Pupae	Total
0	N larvae/dip	2.93	0.56	0.07	3.56	3.02	0.73	0.04	3.80	7.2	8.2	0.7	16.1	16.6	7.8	2.2	26.6
1	N larvae/dip	1.69	0.70	0.02	2.41	1.21	0.04	0.02	1.27	8.0	7.2	1.3	16.5	1.4	2.2	0.5	4.1
	**DR (%)**					**31**	**95**	**0**	**53**					**92**	**68**	**88**	**85**
2	N larvae/dip	3.36	0.90	0.08	4.33	2.0	0.2	0.00	2.3	6.5	7.4	0.9	14.8	1.9	1.2	0.3	3.4
	**DR (%)**					**42**	**82**	**100**	**53**					**88**	**83**	**88**	**86**
3	N larvae/dip	4.00	1.38	0.05	5.43	1.93	0.51	0.00	2.45	6.2	5.8	0.8	12.8	2.8	1.3	0.3	4.4
	**DR (%)**					**54**	**73**	**100**	**60**					**81**	**76**	**90**	**79**
7	N larvae/dip	3.02	2.12	0.06	5.20	3.68	3.03	0.17	6.89	5.5	6.6	0.7	12.8	4.3	2.6	0.2	7.1
	**DR (%)**					**0**	**0**	**0**	**0**					**66**	**59**	**90**	**66**
10	N larvae/dip	2.54	2.09	0.29	4.92	3.77	3.26	0.31	7.34	5.2	4.7	1.6	11.5	4.7	3.4	0.8	8.9
	**DR (%)**					**0**	**0**	**0**	**0**					**61**	**26**	**83**	**53**
13	N larvae/dip	–	–	–	–	–	–	–	–	5.9	5.4	1.5	12.8	7.7	5.4	1.7	14.9
	**DR (%)**					–	–	–	–					**44**	**0**	**62**	**29**
16	N larvae/dip	–	–	–	–	–	–	–	–	4.3	4.9	1.4	10.5	10.8	6.9	1.8	19.5
	**DR (%)**					–	–	–	–					**0**	**0**	**57**	**0**

*RD = Density reduction.*

**Figure 2:**
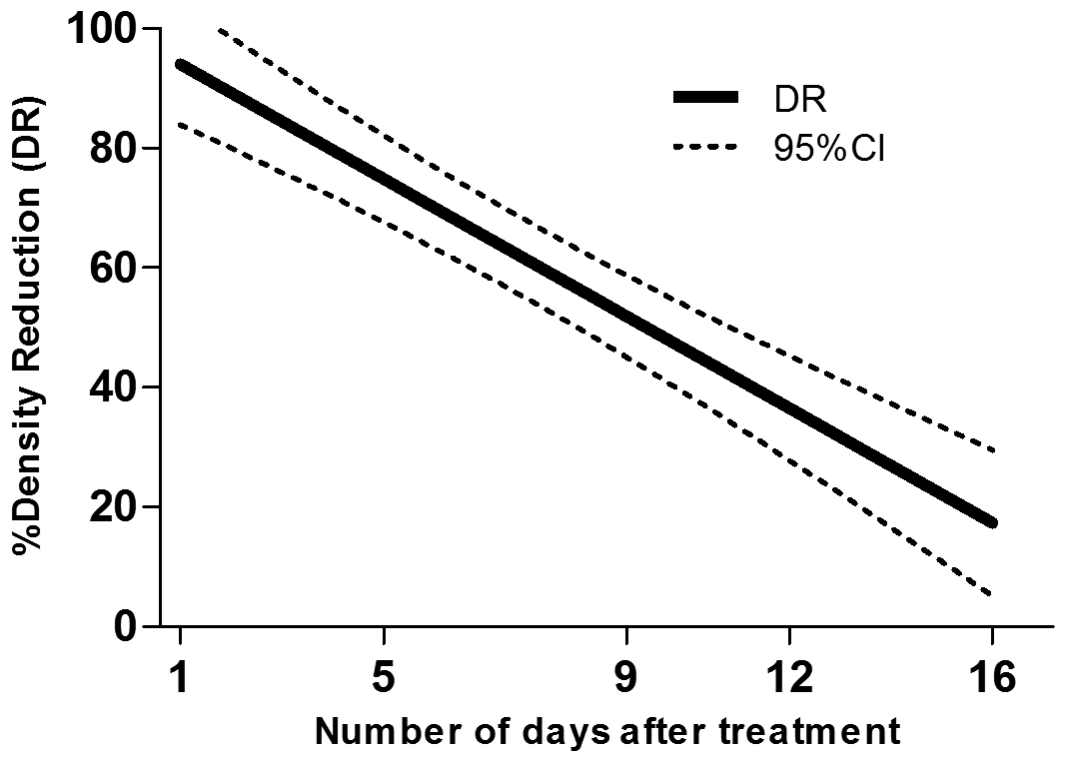
Density reduction (DR) of *Cx. quinquefasciatus* young instars larvae estimated by the regression model according to the number of days after treatment.

**Figure 3:**
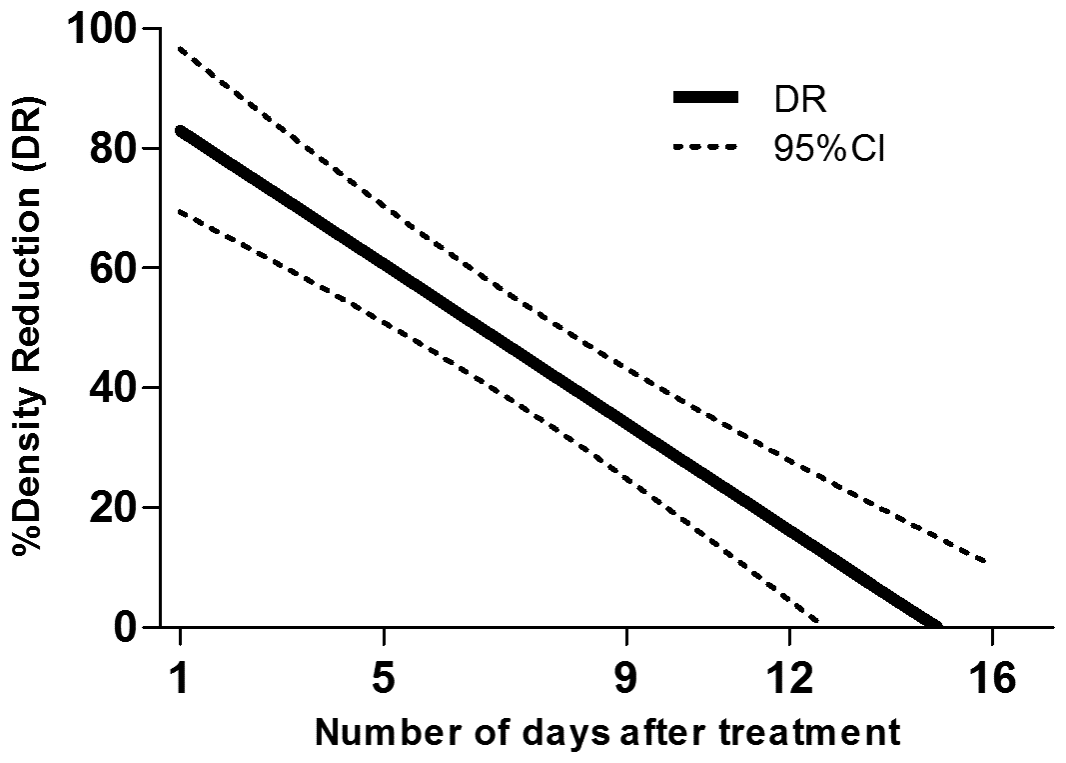
Density reduction (DR) of *Cx. quinquefasciatus* old instars larvae estimated by the regression model according to the number of days after treatment.

**Figure 4:**
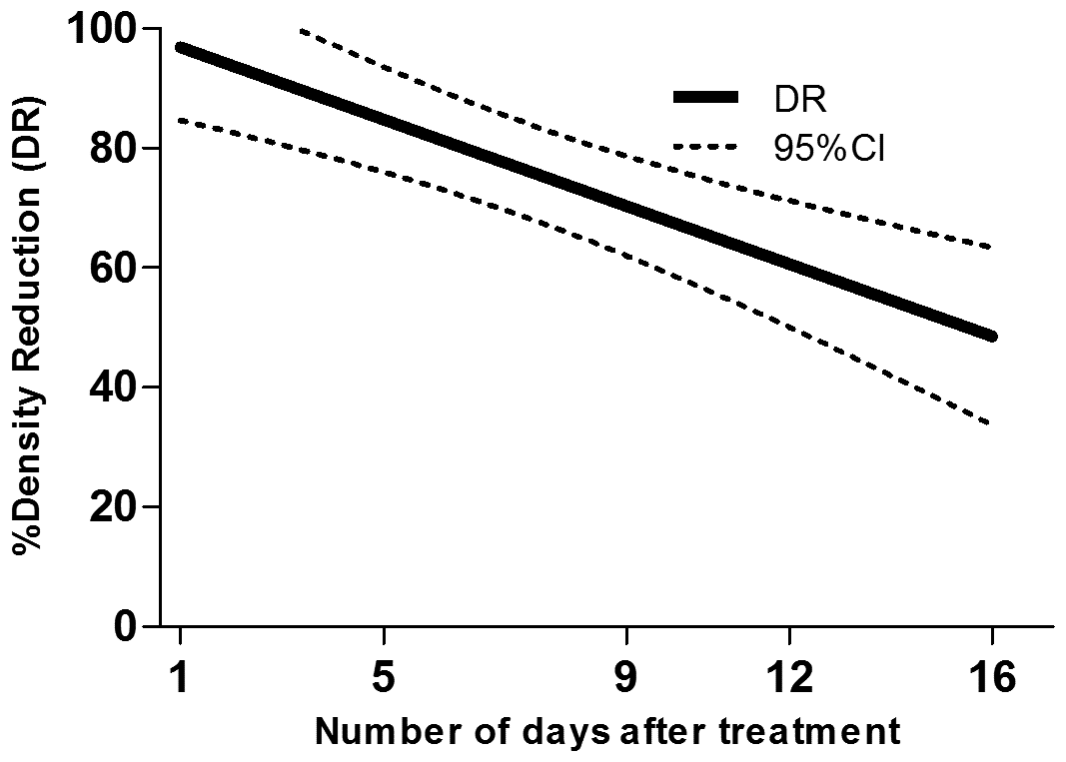
Density reduction (DR) of *Cx. quinquefasciatus* pupae estimated by the regression model according to the number of days after treatment.

The densities of early instars, late instars and pupae of *Cx. quinquefasciatus* in control cesspits before treatment were 7.2 per dip, 8.2 per dip and 0.7 per dip respectively. In the cesspits to be treated the densities were 16.6 per dip, 7.8 per dip and 2.2 per dip respectively. After treatment with Vectobac GR, >80% reduction was observed until day 3 in early instars, until 2 days in late instars and until day 10 in pupae (Table 3). According to the linear regression model, the estimated numbers of days after which the density of late instars larvae would be reduced by 80% (DR_80_) and 50% (DR_50_) were 2 days (0–4) and 7 days (5–8), respectively. DR_80_ and DR_50_ were 4 days (2–5) and 10 days (8–11), respectively for early instars larvae and 6 days (4–9) and 16 days (11–20), respectively for pupae (Figures 2, 3 and 4).

## Discussion and Conclusions

The efficacy of Vectobac GR, a new formulation of *Bacillus thuringiensis var. israelensis* Strain AM65-52, was evaluated against *An. gambiae* and *Cx. quinquefasciatus* in both simulated and natural conditions.

Under simulated field conditions, Vectobac GR caused emergence inhibition of ≥80% up to 21 days (20–22) post-treatment for *An. gambiae* (at 1.2 g/m^2^) and 28 days (27–29) for *Cx. quinquefasciatus* (at 2 g/m^2^). The longer efficacy of Vectobac GR against *Cx. quinquefasciatus* can be explained by the higher dosage of Vectobac GR used during the trial (as per manufacturer’s recommendation) and/or by a higher susceptibility of *Cx. quinquefasciatus* larvae to *Bti* as reported elsewhere [14], [15].

In the field, Vectobac GR formulation, designed for deep penetration of overgrown vegetation after application, induced a ∼50% reduction of *An. gambiae* density in rice fields 3 days after application. The reduction of *Cx. quinquefasciatus* larvae was about 80% in urban cesspits 3 days after application. The short residual efficacy against both *Anopheles* and *Culex* mosquitoes in open water bodies may be due to its low ITU content (200 ITU/mg compared with previous *Bti* formulations with 3000 ITU/mg) or a faster degradation or sequestration of *Bti* toxins in natural habitats as previously reported [16].This short residual efficacy of Vectobac GR in natural habitats inevitably rose the question about the bioavailability of the *Bti* toxins in the rice field ponds and highly polluted habitats such as cesspits. *Bti* toxins are known to sediment in the breeding sites and this is also true with the Vectobac GR (granules were found at the bottom of the cement containers in simulated field conditions). In rice fields, the targeted species population (*An. gambiae s.s.)* was exclusively made of the M molecular form (recently renamed *Anopheles coluzzii*) [17]. Compared to the S molecular (now *Anopheles gambiae*) form, the M form larvae of are known to spend significantly more time at the bottom of the water column in breeding sites to collect food than the S form larvae [18]. Consequently, it is unlikely that the sedimentation of the Vectobac GR toxins might have caused lower control of M form *An. gambiae*. In contrast, we observed that in the water ponds, the granules were sometimes found buried in the mud. Between the granule sp, thus were not fully available for mosquito control.

In addition, it is likely that the direct sunlight exposure of the larval habitats contributed to reduce the residual efficacy of Vectobac GR. Regarding *Culex quinquefasciatus*, results obtained in this present study are consistent with previous trials conducted in polluted (stagnant) waters in Africa [19] and India [20]. The presence of debris and heavy load of organic materials in the cesspits are known to absorb the *Bt* toxins and hence reduce the performance of *Bti*-based products.

With the development and rapid spread of insecticide resistance in malaria vectors [21] and increased proportion of malaria vectors that feed outdoors in response to the implementation of vector control intervention such treated nets and indoor residual spraying [22], [23], there is a urgent need for complementary vector control strategies that could better impact vector density and malaria transmission. As suggested by Corbel et al. [24], the use of larvicide products could be complementary tool in the context of an integrated vector management for malaria transmission reduction and vector resistance management.

The use of larvicidal products for malaria control has long history in Africa with however more or less success [6]. In Gambia, hand application of water-dispersible granular formulations of *Bti* (Valent BioSciences, USA) to water bodies was associated with a 88% reduction in larval densities but had no effect on adult mosquito density and clinical malaria [25]. It is essential to better assess the impact of larviciding on mosquito density, malaria transmission and malaria morbidity. The opportunity to reinforce the use of larviciding in public health is currently under the spotlights among African countries through Economic Community of West Africa States (ECOWAS) including Benin [26]. This can also be an option to manage the spread of pyrethroid-resistance in African malaria vectors, as well as complement control of lymphatic filariasis in Africa south of Sahara. Nevertheless this complementary tool is highly dependent of the larval breeding site dynamics. The cost-effectiveness of such vector control strategy should be also carefully investigated. The present results emphasize the crucial need to improve basic knowledge on mosquito ecology as well as precise identification, mapping and monitoring of larval habitats in order to enhance the public health benefit to implement larval control programs.
